# Household fuel use, smoking and prevalence of self-reported allergic rhinitis in university students in Palestine: a cross-sectional study

**DOI:** 10.3389/falgy.2024.1492213

**Published:** 2024-11-20

**Authors:** Nuha El Sharif, Lana Hnaihen

**Affiliations:** Faculty of Public Health, Al Quds University, Jerusalem, Palestine

**Keywords:** allergic rhinitis, exposures, environmental, prevalence, risk factors

## Abstract

**Purpose:**

In Palestine, few studies investigated the prevalence of allergies and the factors associated with their occurrence. An online survey was conducted on health complex University students in Jerusalem to determine the prevalence of allergy rhinitis (AR) and its relationship with indoor environmental exposures.

**Methods:**

This study employed a modified online Google form of the Global Asthma Network's Adult Questionnaire. The data were reported as frequency and percentage. The chi-square test of independence was performed to investigate the association between AR diagnosis and other factors. Multivariable models were used to identify the independent risk factors for AR after adjusting for potential confounders.

**Results:**

Data was collected from a total of 819 participants. The mean age of the participants was 20 ± 2 years and 78.1% (*n* = 640) were females. The AR diagnostic rate was 10.3%. In addition, having asthma and eczema were found to be substantially associated with AR. Additionally, a family history of AR and other allergens were major predictors of AR. The findings revealed that utilizing animal dung for heating increased the likelihood of AR fourfold (AOR = 4.870, *p*-value = 0.004), whereas e-cigarette vaping increased the possibility of AR by 2.5 times. However, using natural gas for cooking was not significantly associated with AR, and participant age was only slightly associated with AR diagnosis.

**Conclusions:**

Our study found that the AR prevalence rate is low when compared to the same population in other countries. Genetics, biomass fuel consumption, and e-smoking are all significant risk factors for AR in Palestine. An awareness campaign must be developed to educate university students and the general public about the risks of smoking, indoor air pollution, respiratory disorders, and AR. Longitudinal research is required to discover whether these associations are only transient.

## 1 Introduction

Hay fever, a term often used for seasonal allergic rhinitis, is characterized by inflammation of the nasal mucosa, which causes symptoms such as sneezing and congestion. Allergic rhinitis (AR) severity is often underestimated since it is not life-threatening. However, it can cause severe morbidity, both physically and in terms of quality of life and well-being (1).

Research suggests that the prevalence of allergic rhinitis (AR) among adults is increasing in developing countries. According to Al-Digheari et al. (2), the prevalence rate of AR is 32% in the United Arab Emirates, 3.6% in Egypt, and 6.4% in the Gulf cluster (2). In Turkey, the diagnosed prevalence of AR among university students was 15.9% (3). Almalki et al. (4) reported a diagnosis of AR in 39.9% of Saudi Arabia's medical students (4). Similarly, a study by Seedat et al. (5) found that the diagnosed prevalence of AR among medical students in South Africa was 39.1% (5).

Evidence suggests that both genetics and environment contribute to allergy disorders, including allergic rhinitis (6). Genetic factors are unlikely to explain the rapid increase in the incidence of these disorders. Hence, environmental factors have a role in the increased rate of allergic disorders. Studies (7–10) have linked indoor air pollution, including multiple pollutants, to increased allergy risk. Environmental tobacco smoke (ETS) from primary and secondhand smoke remains an important source of household air pollution. Furthermore, scientific research conducted by Chung et al. (11) and Martinasek et al. (8) has demonstrated a clear association between the use of tobacco products, specifically cigarette smoking, and the development of allergic rhinitis and asthma (8, 11).

Biomass fuels, including wood, dung, agricultural crop waste, and coal, are significant factors in indoor air pollution, especially in developing countries (9). Research has also indicated that traditional methods of heating and cooking can increase pollution levels in homes that do not use solid fuels (7, 9, 12). In contrast to the harmful effects of biomass fuels, the use of cleaner alternatives like natural gas and propane has shown promise in reducing respiratory health risks. These fuels release significantly less particulate matter (PM) like PM_2.5_ and nitrogen oxide (NO_2_) than solid fuels, resulting in improved indoor air quality and potentially fewer respiratory problems (7, 12, 13). A recent systematic study and meta-analysis conducted by Puzzolo et al. reported that switching from polluting fuels to gaseous household fuels could reduce health risks, related morbidity, and mortality in countries with limited resources and the highest reliance on polluting fuels. Despite the slightly elevated risk of numerous health outcomes associated with gas fuel usage compared to electricity, it remains a crucial interim option for areas where reliable power for cooking or heating is unattainable (13).

Palestine has conducted limited research on AR. A study among Palestinian university students revealed a 3.1% prevalence of allergic rhinitis (14). However, no recently published research in Palestine investigated the association between the type of fuel used for cooking and heating and the occurrence of allergies. Therefore, this study aimed to determine the prevalence of AR and its association with exposure to household fuel and smoking types among Health Complex University students at Al Quds University, Jerusalem, Palestine.

## 2 Material and methods

### 2.1 Study population

The study population was determined as students studying at Al Quds University Health Complex from September to November 2023. The total number of registered students at the health complex was 6,870 students. About 65% of students in the Health Complex are females (15).

### 2.2 Study design

Observational studies are a key method in research, particularly when performing experiments that are not feasible or unethical. This study utilized a descriptive design, which is a cross-sectional study design. This research design is suitable for performing population-based studies to estimate prevalence and identify risk variables. Moreover, it is advantageous for planning, monitoring, and evaluating public health measures.

### 2.3 Setting

This study was conducted at Al-Quds University Health Complex. The health complex consisted of five faculties (Faculty of Allied Health Profession, Faculty of Dentistry, Faculty of Medicine, Faculty of Pharmacy, and Faculty of Public Health).

### 2.4 Sampling and sample size

A two-stage stratified random sampling procedure based on faculty and year of study was utilized. We distributed the sample size using a probability proportional to the faculty number of students. All faculty's classes were selected at random from each academic year. We selected the sampling class from the course list based on aspects such as the days of the week, faculty, and academic year. During the lecture, the researcher provided a link to the questionnaire for students to complete.

Using the EPI tools program, the sample calculation formula yielded 385 students to be sampled using the following parameters: a precision level of 95 percent, and an estimated percentage level of the allergic rhinitis variable of 5%. The expected response rate was 80%, so we added another 20% to the calculated sample size. In total, the targeted sample size was 461.

### 2.5 Study tool

Data were collected through a structured, self-administered questionnaire that was nationally validated following the recommendations of the Global Asthma Network (GAN) procedures (16). GAN Phase I adult questionnaire (16); a translated adult's questionnaire to Arabic language, was used to collect information that answers the study questions (17). It is a standardized questionnaire provided by the ISAAC and developed by the GAN. These questions are sensitive, specific, and have high predictive validity (16). The questionnaire used to diagnose allergic rhinitis included the following questions: “Was your hay fever confirmed by a doctor?” and, “Have you ever had hay fever?”.

The questionnaire consisted of four sections. The first section included the personal history of allergies and the family history of atopy questions. The second section covers questions about domestic fuel used for heating and cooking, the location of cooking at home, and how ventilation is handled while cooking. The third section comprises questions about university students’ behaviors and lifestyles. The fourth section contained questions about students’ socio-demographic characteristics such as age, gender, marital status, place of residence, and faculty type, as well as their height and weight at the time of the survey. During the investigation, two new definitions of domestic fuel type were developed. Biomass fuel was defined as the use of coal/lignite, charcoal, wood, straw/grass/shrubs, animal dung, and agricultural crop residue. Modern fuel is described as the use of natural gas, biogas, kerosene, and liquefied petroleum gas. Participants have the option to select multiple fuel sources for cooking and heating, if applicable.

The data collection process employed an electronic link from Google Forms. After receiving authorization from the faculty dean and the class instructor, this link was shared with each class's “WhatsApp group” during class hours.

### 2.6 Statistical analysis

Data was entered and analyzed using IBM SPSS Statistics (version 25). Frequencies, means, and standard deviation were used to describe the study data. Bivariate analysis was used to study the relationship between dependent and independent variables. The *p*-value < 0.05 was considered significant with a 95% confidence interval. Multivariate analysis was done using logistic regression to eliminate the effect of cofounders. The logistic model includes all variables of interest to be included in the model. Adjusted odds ratio and 95% confidence interval are reported.

In the analysis, we introduced two new variables. The total biomass fuel variable comprises fuel types such as coal/lignite, charcoal, wood, straw/grass/shrubs, animal dung, and agricultural crop residue. In contrast, the total modern fuel variable includes the use of natural gas, biogas, kerosene, and liquefied petroleum gas. Two variables were created for the cooking and heating methods of fuel utilization.

### 2.7 Ethical considerations

The methods used in this study complied with the Declaration of Helsinki. Al Quds University's Ethical Review Committee approved it. This electronic survey was conducted anonymously. The study questionnaire began with written information regarding its objective and how the data will be used. When students completed the questionnaire, they gave informed consent for participation in this study.

## 3 Results

### 3.1 Participants’ characteristics and allergic rhinitis

Data was collected from a total of 819 participants. The mean age of the participants was 20 ± 2 years; 21.9% (*n* = 179) were males, and 78.1% (*n* = 640) were females. While the rate of those who thought they had ever had allergic rhinitis was 20.8% (*n* = 170), the rate of those diagnosed by a doctor was 10.3% (*n* = 84). Table 1 shows the association between the study's socio-demographics and AR. None of the socio-demographics had significant differences with diagnosed allergic rhinitis.

**Table 1:** Associations between the study's socio-demographics and AR.

		Total	Diagnosis		No diagnosis		Chi-squared	*P*-value
		*N* = 819	*N* = 84		*N* = 735			
		*N*	*N*	%	*N*	%		
Faculty	Allied profession	221	18	21.4%	203	27.6%	3.05	0.55
	Dentistry	118	12	14.3%	106	14.4%		
	Medicine	221	26	31.0%	195	26.5%		
	Pharmacy	200	24	28.6%	176	23.9%		
	Public Health	59	4	4.8%	55	7.5%		
Academic year	First	162	15	17.9%	147	20.0%	9.11	0.11
	Second	247	21	25.0%	226	30.7%		
	Third	159	19	22.6%	140	19.0%		
	Forth	138	10	11.9%	128	17.4%		
	Fifth	80	12	14.3%	68	9.3%		
	Sixth	33	7	8.3%	26	3.5%		
Gender	Male	179	18	21.4%	161	21.9%	0.010	0.92
	Female	640	66	78.6%	574	78.1%		
Marital status	Not single	32	5	6.0%	27	3.7%	1.04	0.31
	Single	787	79	94.0%	708	96.3%		
Place of residence	City	460	48	57.1%	412	56.1%	0.46	0.92
	Village	301	30	35.7%	271	36.9%		
	Refugee Camp	36	3	3.6%	33	4.5%		
	Other	22	3	3.6%	19	2.6%		

Table 2 displays significant associations between the history of diagnosed asthma, asthma ever, and wheezing in the past year (*p*-value < 0.001) and eczema and having eczema ever (*p*-value = 0.004) and being diagnosed with AR. Also, being from a family with a history of asthma, eczema, and rhinitis was significantly associated with being diagnosed with AR (*p* < 0.001). In addition, those who reported smoking (tobacco or waterpipe), those who reported using e-cigarettes, or both (e-cigarettes and smoking) had significant associations with AR too (*p* < 0.05). However, obesity did not have any significant association with AR.

**Table 2:** Associations between students’ comorbidities, family history, and AR.

		Total	No self-reported AR		Self-reported AR diagnosis		Chi-squared	*P* value
		*N* = 819	*N* = 735		*N* = 84			
		*N*	*N*	%	*N*	%		
Wheezing past year	Yes	172	134	18.2%	38	45.2%	33.1	<0.001
	No	647	601	81.8%	46	54.8%		
Asthma ever	Yes	82	56	7.6%	26	31.0%	45.5	<0.001
	No	737	679	92.4%	58	69.0%		
Eczema ever	Yes	189	159	21.6%	30	35.7%	8.40	0.004
	No	630	576	78.4%	54	64.3%		
Have eczema	Yes	129	103	14.0%	26	31.0%	8.40	0.004
	No	364	632	86.0%	58	69.0%		
Have asthma	Yes	51	32	4.4%	19	22.6%	43.80	<0.001
	No	768	703	95.6%	65	77.4%		
Family member asthma	Yes	198	163	22.2%	35	41.7%	15.60	<0.001
	No	621	572	77.8%	49	58.3%		
Family member rhinitis	Yes	191	132	18.0%	59	70.2%	115.20	<0.001
	No	628	603	82.0%	25	29.8%		
Family member eczema	Yes	295	255	34.7%	40	47.6%	5.50	0.019
	No	524	480	65.3%	44	52.4%		
Smoke tobacco or waterpipe	Yes	156	56	7.6%	9	10.7%	4.20	0.040
	No	663	679	92.4%	75	89.3%		
Use e-cigarette	Yes	80	63	8.6%	17	20.2%	11.60	0.001
	No	739	672	91.4%	67	79.8%		
Mixed smoking (e-cigarette and smoking)	Yes	172	145	19.7%	27	32.1%	7.00	0.008
	No	647	590	80.3%	57	67.9%		
Body Mass Index	Overweight/obese	227	198	27.0%	29	34.9%	2.30	0.127
	Normal		535	73.0%	54	65.1%		

Tables 3, 4 display the associations between the use of the various fuels that are used for heating and cooking. In general, all those diagnosed with AR heated their houses in cold weather compared to those without AR (*p*-value = 0.031), and most of those with AR (95.2%) use modern heating systems (electricity, gas, biogas, and Kerosene and liquefied petroleum gas). Also, 32.1% of those with AR use biomass for heating too. These results were significantly different when compared to those without AR (*p*-value = 0.047, and *p*-value 0.005, respectively) (Table 3). In addition, significant associations were shown by using wood, animal dung, and crop residue for heating with having a diagnosis with AR compared to those without (*p*-value < 0.005). However, none of these associations were associated with using any fuel for cooking, the type of stove, the method of ventilation, and the place of cooking (*p*-value > 0.05) (Table 4).

**Table 3:** Associations between AR and the type of fuel daily used in the household for heating.

		Total	No self-reported AR		Self-reported AR diagnosis		Chi-Squared	*P*-Value
		*N* = 819	*N* = 735		*N* = 84			
		*N*	*N*	%	*N*	%		
Do you heat your house when it is cold?	Yes	780	696	94.7%	84	100.0%	4.680	0.031
	No	39	39	5.3%	0	0.0%		
Type of fuel mainly used for heating								
Modern fuel for heating^b^	Yes	727	647	88.0%	80	95.2%	3.93	**0**.**047**
	No	92	88	12.0%	4	4.8%		
Biomass fuel for heating^a^	Yes	168	141	19.2%	27	32.1%	7.77	**0**.**005**
	No	651	594	80.8%	57	67.9%		
Heating by electricity	Yes	671	598	81.4%	73	86.9%	1.57	0.2
	No	148	137	18.6%	11	13.1%		
Natural gas	Yes	273	241	32.8%	32	38.1%	0.96	0.33
	No	546	494	67.2%	52	61.9%		
Biogas	Yes	24	21	2.9%	3	3.6%	0.14	0.71
	No	795	714	97.1%	81	96.4%		
Kerosene	Yes	22	17	2.3%	5	6.0%	3.82	0.05
	No	797	718	97.7%	79	94.0%		
Charcoal	Yes	15	12	1.6%	3	3.6%	1.58	0.21
	No	804	723	98.4%	81	96.4%		
Liquefied petroleum gas	Yes	45	41	5.6%	4	4.8%	0.09	0.76
	No	774	694	94.4%	80	95.2%		
Coal/lignite	Yes	25	20	2.7%	5	6.0%	2.66	0.10
	No	794	715	97.3%	79	94.0%		
Wood	Yes	157	132	18.0%	25	29.8%	6.78	**0**.**009**
	No	662	603	82.0%	59	70.2%		
Straw/shrubs/grass	Yes	44	34	4.6%	10	11.9%	7.86	**0**.**005**
	No	775	701	95.4%	74	88.1%		
Animal Dung	Yes	17	11	1.5%	6	7.1%	11.82	**0**.**001**
	No	802	724	98.5%	78	92.9%		
Agricultural crop residue	Yes	24	18	2.4%	6	7.1%	5.84	**0**.**016**
	No	795	717	97.6%	78	92.9%		

Bold: for variable with *P*-values <0.05.

^a^ Biomass fuel: coal/lignite, charcoal, wood, straw/grass/shrubs, animal dung, and agricultural crop residue.

^b^ Modern fuel: natural gas, biogas, kerosene, and liquefied petroleum gas.

**Table 4:** Associations between AR and the type of fuel mainly used for cooking in the household and ventilation.

		Total	No self-reported AR		Self-reported AR diagnosis		Chi-Squared	*P*-Value
		*N* = 819	*N* = 735		*N* = 84			
		*N*	*N*	%	*N*	%		
No food cooked at home	Yes	75	64	8.7%	11	13.1%	1.74	0.18
	No	744	671	91.3%	73	86.9%		
Modern fuel for cooking^b^	Yes	781	698	95.0%	83	98.8%	2.52	0.11
	No	38	37	5.0%	1	1.2%		
Biomass fuel for cooking^a^	Yes	131	114	15.5%	17	20.2%	1.25	0.26
	No	688	621	84.5%	67	79.8%		
Electricity	Yes	572	512	69.7%	60	71.4%	0.11	0.74
	No	247	223	30.3%	24	28.6%		
Natural gas	Yes	708	630	85.7%	78	92.9%	3.28	0.07
	No	111	105	14.3%	6	7.1%		
Coal/lignite	Yes	70	61	8.3%	9	10.7%	0.56	0.45
	No	749	674	91.7%	75	89.3%		
Wood	Yes	88	78	10.6%	10	11.9%	0.13	0.72
	No	731	657	89.4%	74	88.1%		
Straw/shrubs/grass	Yes	36	31	4.2%	5	6.0%	0.54	0.46
	No	783	704	95.8%	79	94.0%		
Animal dung	Yes	22	18	2.4%	4	4.8%	1.54	0.21
	No	797	717	97.6%	80	95.2%		
Agricultural crop residue	Yes	27	22	3.0%	5	6.0%	2.07	0.15
	No	792	713	97.0%	79	94.0%		
Type of stove usually used	Griddle stove	40	38	5.2%	2	2.4%	4.50	0.29
	Open fire	9	7	1.0%	2	2.4%		
	2–3 pot stove	737	660	89.8%	77	91.7%		
	Sunken pot stove	24	23	3.1%	1	1.2%		
	Don't know	9	7	1.0%	2	2.4%		
Smoke removed by the hood or chimney	neither	429	384	52.2%	45	53.6%	0.06	0.97
	Hood	369	332	45.2%	37	44.0%		
	Chimney	21	19	2.6%	2	2.4%		
Place of cooking	In a room used for living/sleeping	23	19	2.6%	4	4.8%	6.08	0.11
	In a separate room used as a kitchen	735	666	90.6%	69	82.1%		
	In a separate building used as a kitchen	52	43	5.9%	9	10.7%		
	Outdoors	9	7	1.0%	2	2.4%		
Type of ventilation present where the stove is used	Closed room	47	40	5.4%	7	8.3%	2.25	0.52
	Room with eaves spaces	37	32	4.4%	5	6.0%		
	Room with open windows/doors	680	615	83.7%	65	77.4%		
	Room with 3 or fewer walls	55	48	6.5%	7	8.3%		

^a^ Biomass fuel: coal/lignite, charcoal, wood, straw/grass/shrubs, animal dung, and agricultural crop residue.

^b^ Modern fuel: natural gas, biogas, kerosene, and liquefied petroleum gas.

### 3.2 Multivariate analysis

Table 5 displays the study's multivariate regression models. In the first model (model 1), total heating by biomass fuel, total heating by modern fuel, total cooking by biomass, total cooking by modern fuel, current tobacco smoking, waterpipe smoking at the place of residence, electronic cigarette smoking, type of ventilation, and place of cooking were included in the model. After controlling for sociodemographic variables (model 1) results revealed that biomass utilized for heating had a significant connection with AR (AOR = 1.995, *p*-value = 0.010). Furthermore, smoking electronic cigarettes increased the likelihood of being diagnosed with AR when compared to those who did not have it (AOR = 2.842, *p*-value = 0.001). Moreover, participant age and AR diagnosis had a weakly significant association.

**Table 5:** Multivariate analysis for fuel type, smoking, participants’ history of asthma and eczema, and familial factors that determine allergic rhinitis (AR).

	Sig.	AOR	95% CI for AOR	
			Lower	Upper
Model 1				
Participants’ age (continuous)	0.004	1.18	1.05	1.33
Biomass fuel used for heating (Yes/No)	0.010	1.99	1.18	3.38
Electronic cigarette use (Yes/No)	0.001	2.84	1.52	5.299
Model 2				
Participants' age (continuous)	0.009	1.16	1.04	1.29
Electronic cigarette use (Yes/No)	0.003	2.53	1.38	4.66
Natural gas used for cooking (Yes/No)	0.054	2.37	0.99	5.71
Animal dung use for heating (Yes/No)	0.004	4.87	1.66	14.28

AOR, adjusted odds ratio; 95% CI, 95% confidence interval; Sig., significance.

Model 1: The logistic regression model includes total heating by biomass fuel, Total heating by modern fuel, total cooking by biomass, Total cooking by modern fuel, current tobacco smoking, water pipe (narghile) smoking at the place of residence, electronic cigarette smoking, type of ventilation, place of cooking, type of stove after controlling for faculty, academic year, age (continuous), gender of the respondent, marital status, place of residence, and Body Mass Index.

Model 2: The logistic regression model includes: all sources of fuel for heating and cooking used separately for each type of fuel, current tobacco smoking, water pipe (narghile) smoking at the place of residence, electronic cigarette smoking, type of ventilation, place of cooking, type of stove, after controlling faculty, the academic year, age (continuous), gender of the respondent, marital status, place of residence, and Body Mass Index.

In Model 2, all sources of fuel for heating and cooking are used separately for each type of fuel, current tobacco smoking, water pipe (narghile) smoking at the place of residence, electronic cigarette smoking, type of ventilation, place of cooking, and type of stove were included in the model. After controlling for study confounders, the findings revealed that utilizing animal dung for heating increased the likelihood of AR fourfold (AOR = 4.870, *p*-value = 0.004), whereas e-cigarette vaping increased the possibility of AR by 2.5 times. However, using natural gas for cooking was not significantly associated with AR, and participant age was only slightly associated with AR diagnosis.

## 4 Discussion

This study found that the prevalence rate of self-reported diagnosed allergic rhinitis among health complex students at Al Quds University aged 18-23 years is 10.3%. Also, 20% of students reported ever having allergic rhinitis symptoms. In Turkey, a study among university students showed that the rate of nasal allergies was 23.8% and the rate of AR diagnosis was 15.9% (3). In another study in Saudia Arabia, 39.9% of medical students reported having AR (4). Also, among medical students in South Africa, the prevalence of AR was 39.1% (5). The rate of Palestinian students is considered relatively low compared to other countries. Various questionnaires were implemented in these countries. Objective assessment was not implemented in our study. Consequently, it is possible that the students who completed this questionnaire were unaware that they had AR. Although 20% of respondents said they had experienced AR symptoms in the past, they have not yet been diagnosed.

In this study, students with a history of diagnosed asthma had triple the probability of having AR (AOR 3.13, *p*-value < 0.001), and those having a diagnosed eczema had doubled probability for AR too (AOR 2.21, *p*-value < 0.005). The association between AR and asthma and eczema has been extensively reported in the literature (18, 19). In this study, only 2.3% (*N* = 19) of students reported having both asthma and AR diagnosis. It was reported that rhinitis is frequently related to asthma, eczema, and atopy. However, these allergic conditions co-occur more in non-atopic patients than would be expected by chance (20). Epidemiological evidence suggests a strong relationship between AR and asthma. AR can occur in 75% of patients with asthma, whereas asthma can affect up to 40% of patients with AR (21–23). There is now accumulating evidence that AR often precedes the onset of asthmatic symptoms (24). A recent review study by Bousquet et al. (25) evaluated the concept of “one-airway-one-disease,” and analyzed the simplistic approach to the connections between upper- and lower-airway allergy disorders (25). The authors of this review found that rhinitis alone (local disease) and rhinitis with asthma multimorbidity (systemic disease) should be considered two different disorders, probably influenced by the microbiome. A longitudinal birth cohort study in eight European countries of 10,107 children between the years 2003 and 2006, found that children with rhinitis and eczema at age 4 had a higher risk of comorbidity at age 8, with IgE sensitization at age 4 being an independent contributor (20).

A family history of allergic rhinitis was a strong determinant for having AR in the study participants (AOR 8.99, *p*-value < 0.001). A study among children aged 6–12 years in Palestine, showed that paternal asthma and maternal hay fever significantly tripled the risk for their children to have wheezing (26). Birth cohort studies have shown that a family history of atopy is a key risk factor associated with increased risk for AR expression (27). In school-age children in Budapest, Hungary, a family history of atopy doubled the risk of the development of AR (28). In China, a recent study by Ren et al. (29), showed that genetic factors, including maternal and paternal rhinitis, asthma, and eczema, were shown to be significantly associated with the prevalence of current rhinitis (29). The complex mechanisms of inheritance, from genetic predisposition of atopy to atopic (allergic) diseases, are still incompletely understood. Some studies suggest that the pathogenesis of allergic diseases is complex and may be caused by a contribution of genetic and environmental factors, especially at the stage of allergen sensitization (6).

In our study, biomass such as coal/lignite, charcoal, wood, straw/grass/shrubs, animal dung, and agricultural crop residue, used for heating increased the risk for AR (AOR = 1.995, *p*-value = 0.010). Also, in another model, only animal dung that is used for heating increased the probability of AR (AOR = 4.870, *p*-value = 0.004). Indoor air pollution, including tobacco smoke, indoor chemical pollutants such as nitrogen dioxide (NO_2_), carbon monoxide (CO), and volatile organic compounds increase the risk for several respiratory diseases (30). Smoke resulting from the combustion of biomass emits a significant quantity of air pollutants that are detrimental to health. These pollutants include respirable particulate matter (PM), carbon monoxide (CO), nitrogen oxides, formaldehyde, benzene, polycyclic aromatic hydrocarbons, and various other hazardous organic chemicals (13, 30). These pollutants are accountable for inducing detrimental health effects such as respiratory tract infections, including asthma and allergic rhinitis, rhino conjunctivitis, chronic obstructive pulmonary disease, and other conditions (30, 31). In rural communities in Kandy, Sri Lanka, using biomass stoves was associated with a higher risk of childhood wheeze (AOR 2.95; 95% CI 1.19–7.33), allergic rhinitis (AOR 3.01; 95% CI 1.42–6.39), and eczema (AOR 7.39; 95% CI 1.70–32.06) compared with households that used clean stoves (32). In South Africa, AR was significantly associated with the use of coal, wood, and kerosene (33). A systematic review and meta-analysis of studies conducted in developed countries (Europe, North America, Australia, and New Zealand) revealed inconclusive and restricted findings regarding the association between exposure to indoor wood-burning and the occurrence of rhinitis and hay fever, as well as the impact of indoor coal-burning on respiratory outcomes in children (34). In our study, biomass is used for heating by 25% of rural residents and 17.9% of urban residents (*p*-value = 0.024). However, the utilization of modern fuels was the same between rural and urban areas. In this study, we found associations between the use of biomass fuel in houses and the prevalence of AR. This behavior or way of life may eventually contribute to persistent respiratory consequences for the exposed individual. It would have been interesting to do indoor air monitoring to supplement our findings and identify what contaminants are prevalent in the atmosphere that may contribute to AR.

In this study, smoking electronic cigarettes (e-cigarettes) doubled the probability of being diagnosed with AR (AOR = 2.38, *p* < 0.05) when compared to individuals who did not have it. The link between conventional cigarette smoking and allergic diseases has been well studied, whereas little research has been conducted on the association of e-cigarettes with allergic diseases. The e-cigarette liquid contains water, vegetable glycerol, propylene glycol, and optional nicotine which is oxidized to form harmful chemicals. These chemicals have been linked to several airway diseases including asthma, and chronic obstructive pulmonary disease (35). Also, e-cigarettes increase the risks of addiction, poisoning, toxicity from inhalation (including seizures), and trauma and burns (36). In addition, there was conclusive or substantial evidence that e-cigarettes can cause indoor air pollution, waste, fires, and the generation of indoor airborne particulate matter (37). In the Korea National Health and Nutrition Examination Survey (38,413 participants) former e-cigarette vapors and current e-cigarettes showed a significantly increased probability for AR compared with never-e-cigarette vapors (38). In our study, 21% smoke cigarettes and e-cigarettes simultaneously, while 10% solely smoke e-cigarettes. Also, our results showed that smoking both modes of smoking increases the risk for AR by 10 times compared to those not smoking at all (crude odds ratio 10.75, 95% confidence interval 6.741–17.14). In a study among university students in the West Bank of Palestine (2023), 19.7% smoke e-cigarettes, and was practiced by 15% among students from the health sciences colleges (39). As a result, multiple use of smoking may have a major negative impact on health due to increased exposure to nicotine and/or toxicants. A systematic review by Banks et al. (37), reported the need for evidence to support policy and regulatory initiatives to minimize e-cigarette use, especially among smokers, children, adolescents, and young adults, and non-smoking cessation objectives. Therefore, smoking e-cigarettes is a potential risk factor for AR. Our study results support what has been reported in similar studies (11, 38, 40). As a result, further research should be conducted into the characteristics and consumption patterns of various tobacco users, as well as their relationship to allergies.

## 5 Study limitations

Although we assumed that the participants had allergic rhinitis, we did not confirm that having allergic rhinitis using allergy tests. We also did not do clinical examinations or allergy tests on those who participated. It is possible that participants had another possible factor causing their chronic rhinitis, although allergic rhinitis is the most common cause of chronic rhinitis (1).

The study is cross-sectional which could hinder the ability to draw causal inferences. However, in contrast to other studies conducted on university students, our sample of health specialist students is adequately representative. This reduces the likelihood of misinterpreting the questions we had on health-related issues. Nevertheless, the study does not encompass the entire population of university students across all faculties or from other universities in different regions of Palestine. Also, this population at the university does not represent the community population in the same age range. In addition, students were asked about the heating and cooking which are practiced at their houses. However, these topics may not be of significant interest to them, and recall bias may be a factor here. We relied on the information of someone who was at home throughout data collection and didn't stay in the university dorms or live alone. Lastly, the data collection started in September when most of the health-related symptoms associated with allergic rhinitis are common due to the dry autumn weather patterns being experienced.

## 6 Conclusion

We found that the prevalence of allergic rhinitis in health complex students at Al Quds University was 10%. This result is considered low compared to the same population in other countries. Also, AR was shown to be strongly determined by having asthma and eczema. Also, family history of AR and other allergies were strong determinants for AR too.

This study is the first in Palestine to examine fuel utilized for heating and cooking and its association with allergic rhinitis. These data indicate that the utilization of fuels for heating affects allergic rhinitis. The findings support the notion that the utilization of household fuel is a contributing factor to respiratory issues, particularly allergic rhinitis. This result can be utilized to raise community awareness in understanding the appropriate ventilation of houses, whether it pertains to cooking or heating with traditional biomass fuels or any modern fuels. Furthermore, its objective is to communicate the possible health advantages of shifting from outdated biomass fuels to new fuel sources, provided they are utilized appropriately.

Another important finding is that e-cigarette is a potential risk factor for AR. An awareness campaign must be initiated to raise the awareness of university students and the community on the negative effects of smoking itself and the alternative electronic smoking and its impact on respiratory diseases and AR. There should be training programs on smoking cessation and smoking ban policies in universities and the public. Further research with a longitudinal study design should be conducted to examine whether these associations are transient.

## Data Availability

The raw data supporting the conclusions of this article will be made available by the authors, without undue reservation.
